# Supplementary material for: The Immune Response in the Uteri and Placentae of Chlamydia abortus-Infected Ewes and Its Association with Pregnancy Outcomes
Source: Pathogens. 2023 Jun 19;12(6):846. doi: 10.3390/pathogens12060846 (PMC10303648; doi:10.3390/pathogens12060846)
Supplement: Supplementary file 1 [file pathogens-12-00846-s001.zip › pathogens-2414565-supplementary.pdf]

Supplementary information

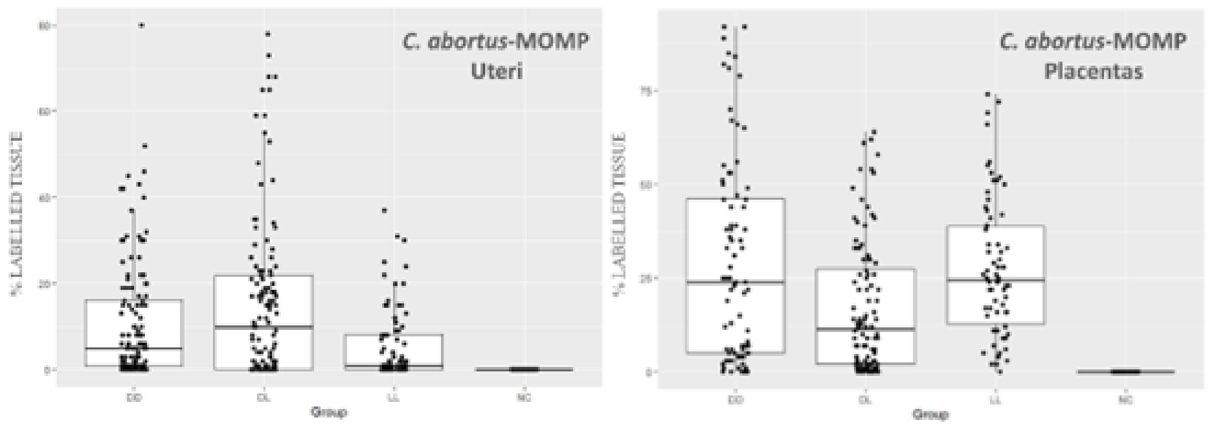

Figure S1. Boxplot showing the immunolabelling for *C. abortus*-MOMP in ovine uteri and placentas. Samples were sorted according to ewe outcomes groups (DD, DL, LL and NC). The boxplot represents the percentage of tissue labelled in each sample using specific *C. abortus*-MOMP IHC. Samples were sorted according to ewe outcomes groups (DD, DL, LL and NC). Boxes in the plots are shown using the first quartile (bottom line of the box), median (central line in the box) and third quartile (upper line of the box). The data used is represented as solid points in the boxplot. Note the difference between NC and the other groups. A statistically significant difference was found between the NC and positive groups (DD, DL and LL), but not among those three groups.

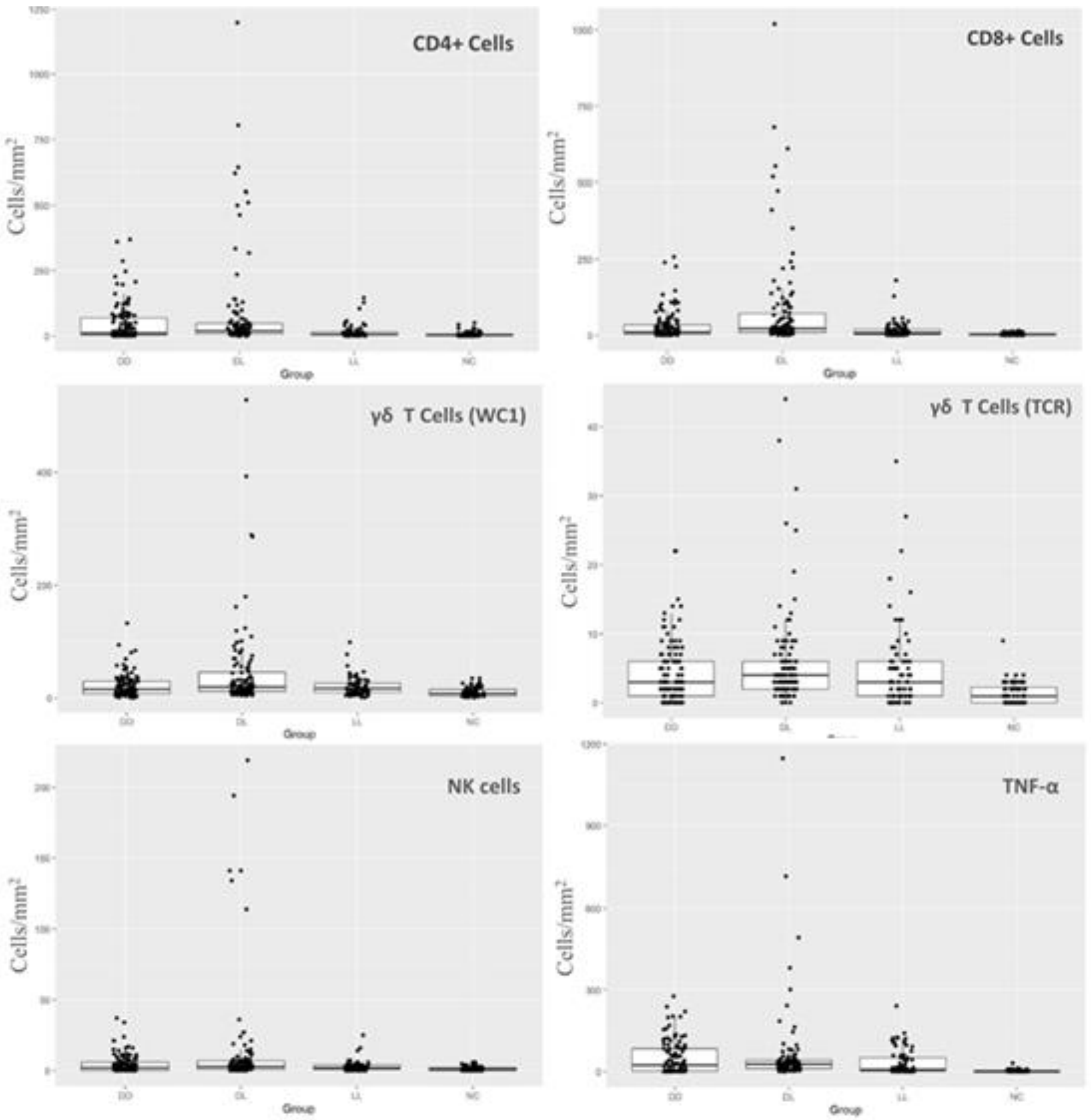

Figure S2. Boxplot showing results for immunolabelling for CD4, CD8, γδ T cells, NK cells and TNF-α in uteri. The boxplot represents the counts of CD4+ T cells in uteri samples labelled using IHC. Samples were sorted according to ewe outcomes groups (DD, DL, LL and NC). Boxes in the plots are shown using the first quartile (bottom line of the box), median (central line in the box) and third quartile (upper line of the box). The data used is shown as a solid point over the standard error bar of the boxplot. No statistically significant effects of the group on the response were found.

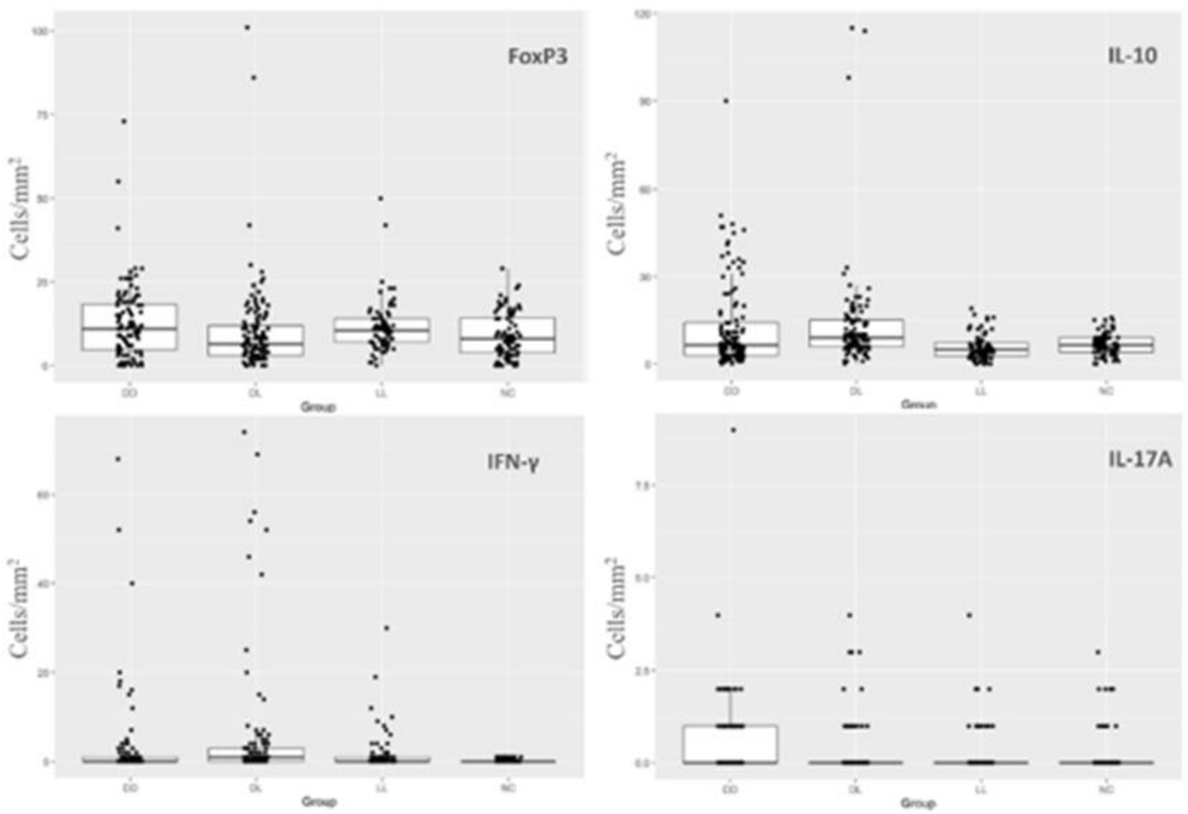

Figure S3. Boxplot showing results for immunolabelling for FoxP3, IL-10, IFN-γ and IL-17A in uteri. The boxplot represents the counts of cells in uteri samples containing positive labelled by IHC using specific mAb (Foxp3 and IL-10) and for ISH using a specific probe (IFN-γ and IL-17A). Samples were sorted according to ewe outcomes groups (DD, DL, LL and NC). Boxes in the plots are shown using the first quartile (bottom line of the box), median (central line in the box) and third quartile (upper line of the box). Data for each group is shown as solid points over the standard error bar of the boxplot. Note the similarity among the boxplots. No statistically significant differences were found.

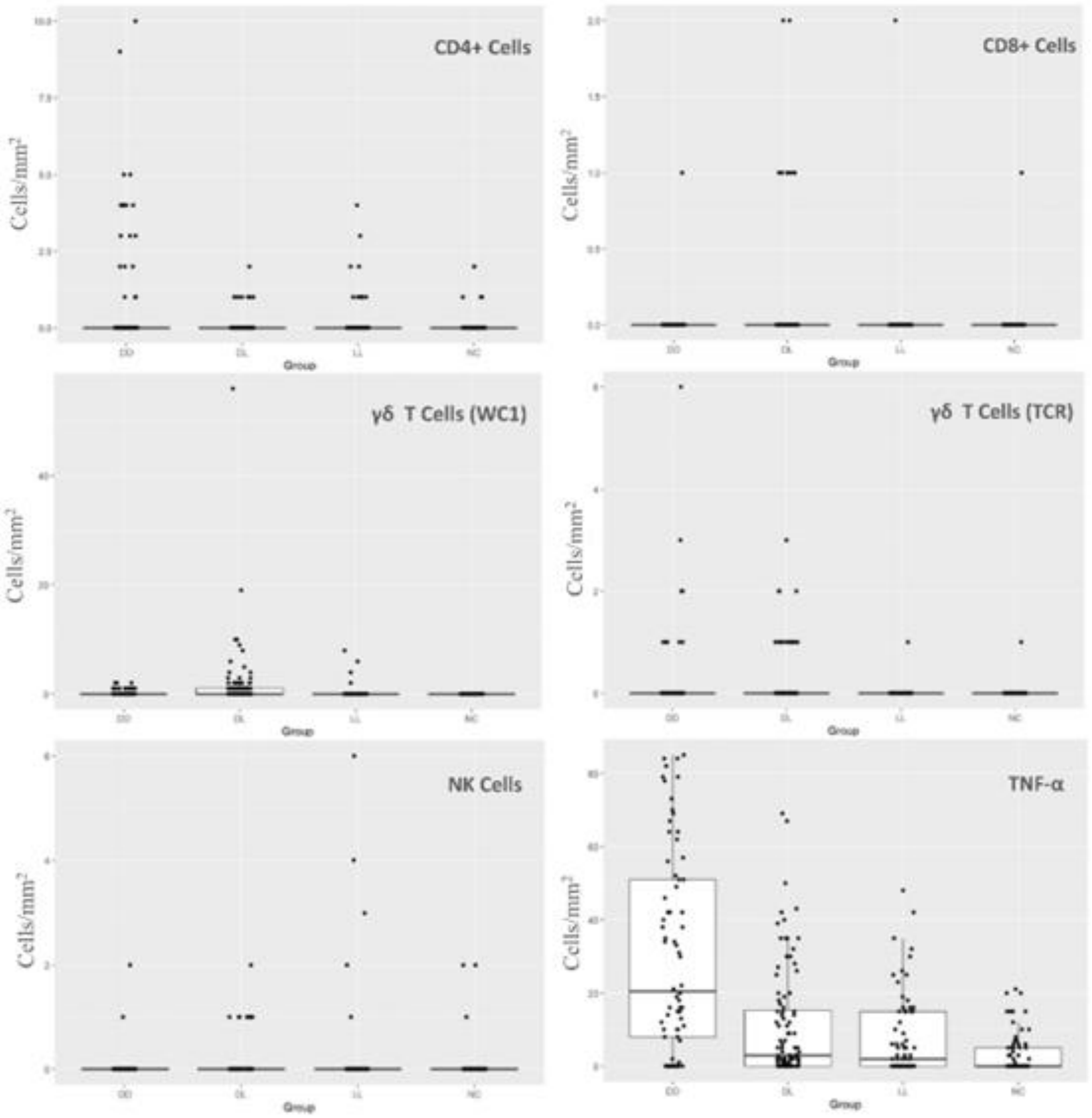

Figure S4. Boxplot showing the summary of the results obtained by IHC-labelling for CD4, CD8, γδ-T cells, NK cells and TNF-α in placentas. The boxplot represents the cell counting in placental samples of cells containing positive labelled by IHC using specific mAb. Samples were sorted according to ewe outcomes groups (DD, DL, LL and NC). Boxes in the plots are shown using the first quartile (bottom line of the box), median (central line in the box) and third quartile (upper line of the box). No sufficient evidence to support a difference in the probability of a zero response between the groups for CD4, CD8 and γδ-T cells or NK cells across the groups.

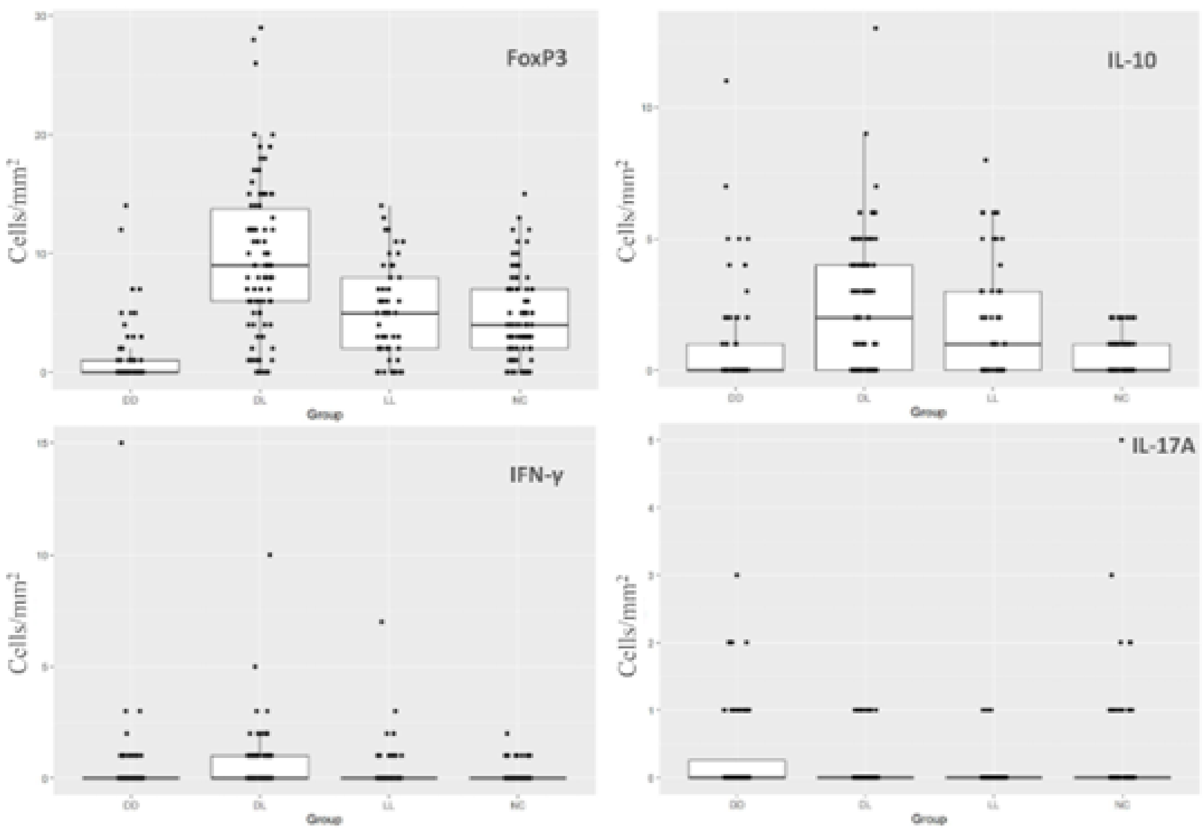

Figure S5. Boxplot showing the summary of the results in the placenta for labelling of Foxp3 and IL-10 by IHC and IFN-γ and IL-17A by IHS. The boxplot represents the counts in placenta samples of cells labelled by IHC or ISH. Samples were sorted according to ewe outcomes groups (DD, DL, LL and NC). Boxes in the plots are shown using the first quartile (bottom line of the box), median (central line in the box) and third quartile (upper line of the box). Corresponding data for each group used for the analysis is shown as solid points over each boxplot. NC group showed statistically significant from the three infection groups by IL-10 and FoxP3, and the DD group were statistically significantly lower than those in the DL and LL. No statistical differences were found between group for IFN-γ and IL-17A.
